# Genetic variants of TRAF6 modulate peritoneal immunity and the risk of spontaneous bacterial peritonitis in cirrhosis: A combined prospective-retrospective study

**DOI:** 10.1038/s41598-017-04895-z

**Published:** 2017-07-07

**Authors:** Martina Mai, Sven Stengel, Eihab Al-Herwi, Jack Peter, Caroline Schmidt, Ignacio Rubio, Andreas Stallmach, Tony Bruns

**Affiliations:** 1 The Integrated Research and Treatment Center for Sepsis Control and Care (CSCC), Jena University Hospital, Friedrich Schiller University of Jena, Jena, Germany; 2 Department of Internal Medicine IV (Gastroenterology, Hepatology, and Infectious Diseases), Jena University Hospital, Friedrich Schiller University of Jena, Jena, Germany; 3 Institute of Molecular Cell Biology, Center for Molecular Biomedicine (CMB), Jena University Hospital, Friedrich Schiller University of Jena, Jena, Germany

## Abstract

Alterations of the innate immunity contribute to the development of spontaneous bacterial peritonitis (SBP) in liver cirrhosis. Given its role in immune signaling, antimicrobial function, and macrophage differentiation, we hypothesized that genetic polymorphisms of *TRAF6* modulate the risk of SBP. Thus, we determined the*TRAF6* haplotype in 432 patients with cirrhosis and ascites using the haplotype-tagging single nucleotide polymorphisms rs331457 and rs5030419. In addition, peritoneal macrophages were immunomagnetically isolated and characterized. Overall, 122 (28%) patients had an episode of SBP. In the combined prospective-retrospective analysis the frequency of SBP differed between the four haplotypes (*P* = 0.014) and was the highest in 102 patients carrying the rs331457 but not the rs5030419 variant, when compared to other haplotypes (odds ratio 1.95 [1.22–3.12]) or to the wild-type (odds ratio 1.71 [1.04–2.82]). This association was confirmed in multivariate logistic regression (adjusted odds ratio 2.00 [1.24–3.22]) and in prospective sensitivity analysis (hazard ratio 2.09 [1.08–4.07]; *P* = 0.03). The risk haplotype was associated with lower concentrations of the immune activation marker soluble CD87 in ascitic fluid and with a decreased expression of IL-6 and CXCL8 in isolated peritoneal macrophages. In conclusion, genetic polymorphisms of *TRAF6* are associated with decreased peritoneal immune activation and an increased risk of SBP.

## Introduction

Spontaneous bacterial peritonitis (SBP) is a frequent infectious complication in decompensated cirrhosis, which occurs in approximately 25% of patients and is associated with significant mortality^1, 2^. Although its pathogenesis is not understood in detail, pathological bacterial translocation from the gut into the circulation accompanied by cirrhosis-associated immune dysfunction has been accepted as the major underlying mechanism^3, 4^. Identified risk factors for SBP either reflect an advanced state of liver disease and portal hypertension or a transient alteration of the gastrointestinal barrier (gastrointestinal hemorrhage)^5^. The finding, that SBP relapses in up to 70% of patients after a first episode without antibiotic prophylaxis^6^ suggests an individual genetic predisposition to SBP in advanced cirrhosis in addition to environmental factors. Multiple lines of evidence link alterations of the innate immunity on the gut mucosal, peritoneal, or systemic level with the risk of SBP. In this context, monocytes and macrophages are crucial for pathogen recognition and bacterial clearance as they express a variety of extra- and intracellular pattern recognition receptors (PRR) to recognize conserved pathogen-associated molecular patterns (PAMPs). Genetic variants associated with PRR signaling, such as in nucleotide-binding oligomerization domain-containing protein 2 (*NOD2*)^7, 8^ and Toll-like receptor 2 (*TLR2*)^9, 10^, have been shown to predispose to SBP. In addition, modulators of TLR signaling, e.g. nuclear dot protein 52 kDa (NDP52)^11^, and immune cell recruitment, e.g. monocyte chemotactic protein 1 (MCP-1)^12^, may play a role in developing SBP in patients with alcoholic cirrhosis.

**Figure 1:**
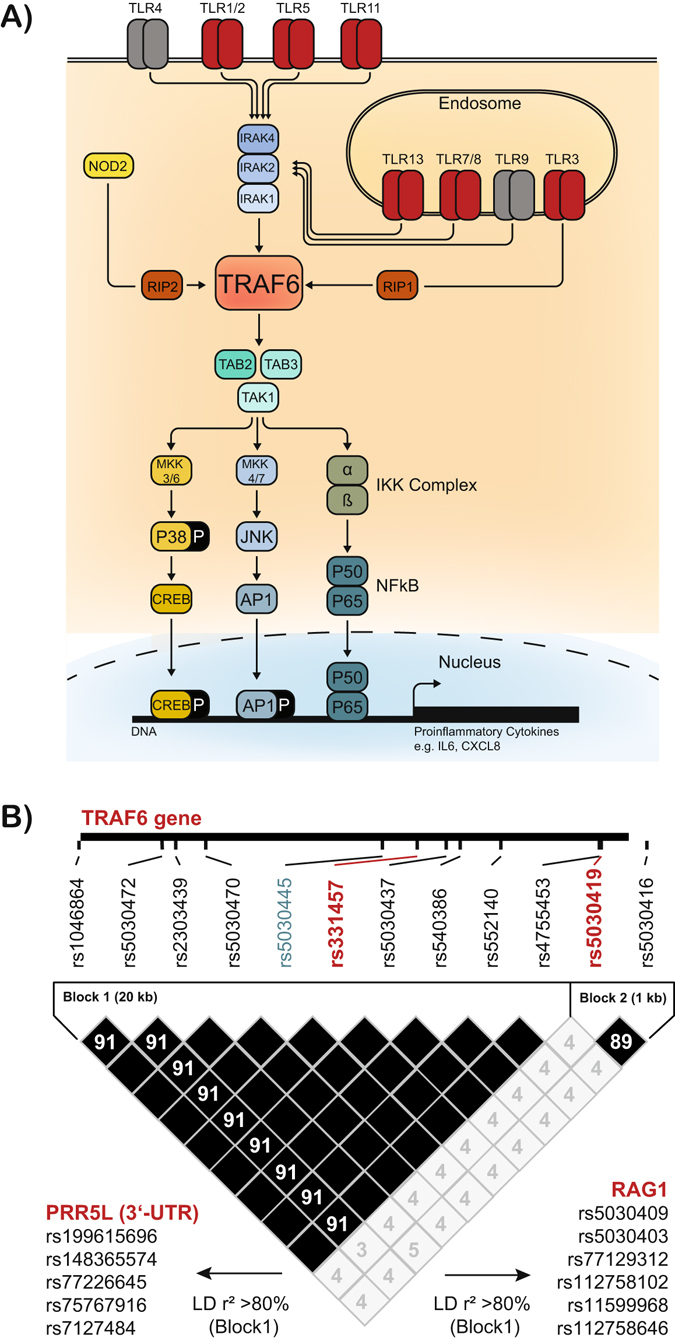
Schematic representation of TRAF6 immune function and *TRAF6* haplotypes. (**A**) Schematic representation showing the role of TRAF6 for Toll-like receptor (TLR) and nucleotide-binding oligomerization domain-containing protein 2 (NOD2) signaling in innate immune cells. (**B**) Linkage disequilibrium (LD) plot with r^2^ values illustrating the TRAF6 gene haplotype definition derived from HapMap data (HapMap release 28 Phase II + III, CEU population, forward strand, http://hapmap.ncbi.nlm.nih.gov) including single nucleotide polymorphisms (SNPs) with a minimum allele frequency of 0.05 and a Hardy Weinberg *P* value cut-off of 0.0010 are shown. The haplotype-tagging SNPs rs331457 and rs5030419 (red) and the validation SNP rs5030445 (blue) are indicated. Furthermore, SNPs in LD with rs331457 in neighboring genes are indicated as identified using HaploReg v4.1.

Tumor-necrosis factor receptor-associated factor 6 (TRAF6), a K63-specific E3 ubiquitin ligase, is a central regulator for controlling myeloid differentiation response gene D88 (MyD88)-dependent signal transduction of TLRs to activate nuclear factor-κB (NF-κB) and to induce proinflammatory cytokine responses^13^. TRAF6 is also activated by NOD2 and therefore necessary to synergistically augment cytokine release after ligation of NOD2 and TLR4^14^. In addition, its activation promotes bacterial killing^15^ and a proinflammatory macrophage phenotype^16^. Owing to its nodal bottleneck position in PRR signaling and its importance in immune activation^17^ (Fig. 1A), we hypothesized a role of germ line polymorphisms of *TRAF6* gene for peritoneal immunity and the development of SBP. We evaluated this hypothesis in a large German cohort of hospitalized patients with decompensated cirrhosis and ascites.

## Results

### Study cohort and follow-up

432 hospitalized patients with cirrhosis and ascites who underwent diagnostic ascitic fluid analysis were included in the combined prospective-retrospective analysis. Patients were predominantly male (71%) and presented with alcoholic liver disease (75%) with a median age of 59 years at inclusion (Table 1

**Table 1:** Patients’ characteristics stratified by the TRAF6 haplotype. Median/IQR or Frequency/Percentage are shown.

rs331457 rs5030419	All patients (N = 432)	Haplotype 1 WT (G) WT (C) (N = 208)	Haplotype 2 MUT (G>A) WT (C) (N = 102)	Haplotype 3WT (G)MUT (C>G)(N = 103)	Mixed HaplotypeMUT (G>A)MUT (C>G)(N = 19)	*P* value*
**Male sex**	308 (71%)	149 (72%)	68 (67%)	78 (76%)	13 (68%)	0.53
**Alcoholic cirrhosis**	325 (75%)	154 (74%)	79 (77%)	78 (76%)	14 (74%)	0.93
***NOD2*** **risk variants** ^**#**^	84 (19%)	43 (21%)	23 (23%)	17 (17%)	1 (5%)	0.29
**Patient characteristics at inclusion**						
**Age**	59 (52–68)	60 (53–68)	58 (47–68)	59 (52–70)	56 (52–66)	0.43
**Hepatocellular carcinoma**	65 (15%)	33 (16%)	10 (10%)	18 (17%)	4 (21%)	0.30
**Child-Pugh stage C**	287 (66%)	140 (67%)	66 (65%)	70 (68%)	11 (58%)	0.80
**MELD**	17 (12–22)	17 (12–22)	18 (12–22)	17 (12–23)	14 (12–17)	0.30
**Ascitic fluid protein (g/l)**	13 (8–20)	13 (9–19)	10 (8–18)	11 (8–21)	21 (9–32)	**0.029**
**SAAG (g/l)**	17 (13–20)	17 (13–20)	17 (13–20)	17 (12–21)	15 (12–15)	0.56
**Bilirubin (µmol/l)**	44 (22–97)	42 (21–98)	46 (26–92)	48 (20–114)	40 (24–64)	0.95
**International normalized ratio**	1.4 (1.2–1.7)	1.4 (1.2–1.7)	1.4 (1.3–1.7)	1.4 (1.2–1.8)	1.4 (1.1–1.6)	0.76
**Creatinine (µmol/l)**	96 (67–148)	90 (67–147)	108 (63–156)	106 (69–148)	81 (60–114)	0.41
**C-reactive protein (mg/l)**	32 (17–60)	32 (16–59)	37 (20–62)	33 (19–57)	22 (12–56)	0.68
**WBC (×10** ^**9**^ **cells/l)**	7.2 (5.0–10.8)	7.0 (5.2–10.0)	6.9 (4.6–12.0)	8.0 (5.5–11.1)	5.7 (3.9–10.2)	0.46
**Platelets (×10** ^**9**^ **cells/l)**	129 (82–185)	137 (87–200)	131 (78–185)	114 (79–181)	90 (69–144)	0.16
**Albumin (g/l)**	24 (20–28)	24 (20–29)	23 (20–28)	25 (20–28)	24 (20–31)	0.94
**Sodium (mmol/l)**	135 (132–139)	135 (131–138)	135 (132–138)	135 (133–139)	136 (132–141)	0.53
**Use of beta blockers**	223 (52%)	100 (48%)	60 (59%)	53 (51%)	10 (53%)	0.36
**Patient characteristics during follow-up**						
**Primary antibiotic prophylaxis at any time** ^**¶**^	71 (17%)	34 (17%)	18 (19%)	17 (17%)	2 (10%)	0.89

^*^P values from from Kruskal Wallis test or Fisher’s exact test as appropriate.

^#^Nucleotide-binding oligomerization domain-containing protein 2 risk variants R702W, G908R and L1007fs.

^¶^Comprising quinolones, cotrimoxazole and rifaximin. Data available from 421 patients (97.5%). Abbreviations: mutation (MUT); wild-type (WT); model for end-stage liver disease (MELD); Serum ascites albumin gradient (SAAG); White blood cell count (WBC); spontaneous bacterial peritonitis (SBP).

). The median model for end-stage liver disease (MELD) score at inclusion was 17 (interquartile range: 12–22) indicating advanced liver disease. Overall, 122 (28%) had at least one documented SBP in the study period including 27 patients with a well-documented history of SBP and 95 patients, who had a first episode of SBP at study entry or developed SBP during the follow-up. Overall, 201 (47%) patients died after a median of 51 days (interquartile 15–175) and 36 (8%) patients received a liver transplant after a median of 120 days (interquartile 19–282). Transplant-free survivors (N = 195) were followed-up for a median of 264 days (interquartile range: 52–823). Twenty-five patients received primary antibiotic prophylaxis with quinolones, cotrimoxazole or rifaximin at inclusion and 46 patients at a later time during follow-up.

### *TRAF6* haplotypes

The *TRAF6* haplotypes were defined using the HapMap release 28 (Phase II + III, CEU population, forward strand, chromosome 11, 36467073–36489113) to identify single nucleotide polymorphisms (SNPs) not being in linkage disequilibrium^18^. As selection criteria a minimum allele frequency of 0.05 and a Hardy Weinberg p-value cut-off of 0.0010 in HaploView 4.2 for each SNP were applied^19^, which resulted in the identification of two haplotype tagging, non-coding single nucleotide polymorphisms (SNPs), rs331457 and rs5030419, covering 12/12 TRAF6 alleles at a mean max r^2^ of 0.984 (Fig. 1B).

The minor allele frequencies of the haplotype-tagging SNPs were 0.16 (139/864 alleles) for rs331457 and 0.15 (126/864 alleles) for rs5030419 as expected in the general population^20^. The most common haplotype 1 (wild type alleles at both tagging loci) was detected in 208 (48%) patients, haplotype 2 (defined by rs331457 mutation G > A and rs5030419 wild-type) in 102 (24%) patients, and haplotype 3 (defined by rs331457 wild-type and rs5030419 mutation C > G) in 103 (24%) patients. Both tagging SNPs were detected in 19 (4%) patients (rs331457 mutation G > A, rs5030419 mutation C > G). These were called mixed haplotype, as they may represent a unique haplotype (both SNPs on one chromosome) or a mix of the haplotypes 2 and 3 (each SNP on a different chromosome). To validate the haplotyping, the allele frequency of the SNP rs5030445 being located in a putative enhancer region within intron 2 of *TRAF6* ^19^ was determined. As predicted (Fig. 1B), linkage between rs5030445 and rs331457 was observed in 406/432 (94%) patients.

### *TRAF6* haplotypes and the risk of SBP

**Figure 2:**
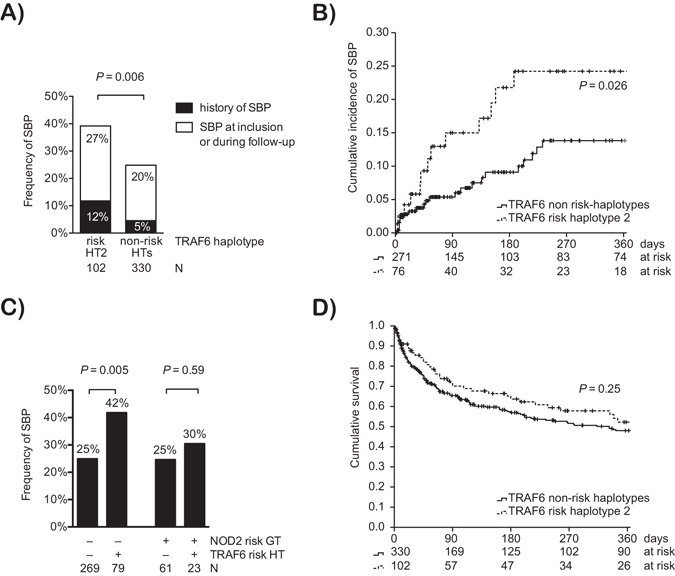
Association of *TRAF6* haplotypes with SBP and survival. (**A**) Combined prospective and retrospective analysis. Frequency of SBP in patients stratified for the presence of the *TRAF6* risk haplotype 2 (HT2) and other haplotypes (non-risk HTs). The frequency of SBP is given for SBP, which were well documented at baseline (history of SBP) and, which were diagnosed at inclusion or during follow-up. *P* value from Fisher’s exact test. (**B**) Prospective analysis. The cumulative incidence of a first episode of SBP in patients without a history of SBP and without SBP at inclusion is indicated and stratified for the presence of the *TRAF6* risk haplotype 2. Data were right-censored at liver transplantation, death, or loss-to-follow-up. Patients at risk and the *P* value from log-rank test are indicated. (**C**) The frequency of SBP in patients stratified for the presence of the *TRAF6* risk haplotype 2 (HT2) and other haplotypes (non-risk HTs) is given for patients carrying at least one of the *NOD2* risk variants R702W, G908R or L1007fs and for patients with the *NOD2* wild-types. *P* values from Fisher’s exact test. (**D**) Kaplan-Meier analysis of cumulative survival of the overall cohort is shown and stratified for the presence of the *TRAF6* risk haplotype 2. Data were right-censored at liver transplantation or loss-to-follow-up. Patients at risk and the *P* value from log-rank test are indicated.

Patients’ characteristics did not significantly differ between the four *TRAF6* haplotypes with exception of ascitic fluid (AF) protein (Table 1). However, there were no differences in the severity liver failure and of portal hypertension between the groups as indicated by surrogate parameters MELD score, serum ascites albumin gradient, and platelet count. In the combined pro- and retrospective analysis, the frequency of SBP significantly differed between the four haplotypes (Table 1) and was the highest in patients with haplotype 2 (Fig. 2A). Among the 347 patients who did not have a history of SBP and did not present with SBP at inclusion, the actuarial cumulative incidence of a first episode of SBP was 16.3% ± 2.7% (standard error) after 1 year according to Kaplan-Meier analysis. In this prospective sensitivity analysis, patients with the *TRAF6* risk haplotype 2 had a significantly higher 1-year cumulative incidence of SBP (24.2% ± 6.0%) as compared to patients without the haplotype 2 (13.8% ± 2.9%; *P* = 0.026 in log rank test) (Fig. 2B) with a hazard ratio for SBP of 2.09 (95% confidence interval [CI]: 1.08–4.07; *P* = 0.03) in presence of the risk haplotype 2.

The unadjusted odds ratio for an episode of SBP in patients with haplotype 2 was 1.71 (95% CI: 1.04–2.82; *P* = 0.036) when compared to patients with the wild-type haplotype, and it was 1.95 (95% CI: 1.22–3.12; *P* = 0.005) when compared to all other patients (Table 2). In univariate analysis higher MELD score and lower albumin at inclusion indicated patients at an increased risk for SBP. After adjustment for MELD and serum albumin, only low serum albumin and the *TRAF6* haplotype 2 remained an indicator for a significantly increased risk of SBP in multivariate logistic regression (Table 2). The *TRAF6*-associated risk for SBP was higher in patients with non-alcoholic cirrhosis (adjusted odds ratio 3.04; 95% CI 1.11–8.36; *P* = 0.031) than in patients with alcoholic cirrhosis (adjusted odds ratio 1.81; 95% CI 1.04–3.17; *P* = 0.037) according the multivariate logistic regression model 2. As the presence of hepatocellular carcinoma (HCC) is associated with higher mortality and shorter follow-up, we also confirmed an association of the *TRAF6* haplotype with SBP for the subgroup of patients without HCC (Supplementary Table S1).

**Table 2:** Risk factors for a first episode of spontaneous bacterial peritonitis.

	Univariate Model		Multivariate Model 1^#^		Multivariate Model 2^#^	
	OR (95% CI)	P value	Adjusted OR (95% CI)	P value	Adjusted OR (95% CI)	P value
***TRAF6***		**0**.**016**	not included	—		**0**.**018**
Haplotype 1	1.00 (reference)				1.00 (reference)	
Haplotype 2	1.71 (1.04–2.82)				1.77 (1.07–2.95)	
Haplotype 3	0.76 (0.44–1.33)				0.78 (0.44–1.38)	
Mixed Haplotype	0.38 (0.07–1.39)				0.35 (0.08–1.57)	
TRAF6 risk haplotype 2*	1.95 (1.22–3.12)	**0**.**005**	2.00 (1.24–3.22)	**0**.**004**	not included	—
*NOD2* risk variant	0.88 (0.51–1.51)	0.64	—	—	—	—
MELD (per 1-point increase)	1.03 (1.00–1.06)	**0**.**046**	1.03 (0.99–1.06)	0.11	1.02 (0.99–1.06)	0.12
Albumin (per 1-g/l increase)	0.95 (0.92–0.99)	**0**.**013**	0.96 (0.92–0.99)	**0**.**038**	0.96 (0.92–0.99)	**0**.**039**
AF protein (per 1-g/l increase)	1.00 (0.98–1.02)	0.84	—	—	—	—
Male Sex	1.00 (0.63–1.59)	1.00	—	—	—	—
Primary antibiotic prophylaxis	0.64 (0.34–1.20)	0.17	—	—	—	—
Beta blocker use at inclusion	1.00 (0.66–1.52)	1.00	—	—	—	—

^#^Binary logistic regression with alternative models including either haplotype overall or the risk haplotype 2 as categorical variable.

*Versus non-risk haplotypes 1, 3, and the mixed haplotype.

^¶^Comprising quinolones, cotrimoxazole and rifaximin. Data available from 421 patients (97.5%).

Abbreviations: odds ratio (OR), confidence interval (CI), tumor necrosis factor receptor-associated factor 6 (TRAF6), nucleotide-binding oligomerization domain-containing protein 2 (NOD2), model for end-stage liver disease (MELD), ascitic fluid (AF).

Of note, the association of the *TRAF6* haplotype with SBP was observed in patients without *NOD2* risk alleles (*P* = 0.005), but not in patients carrying any of the three *NOD2* risk variants R702W, G908R and L1007fs (*P* = 0.59) (Fig. 2C). Among patients with SBP, 20 developed a second episode despite secondary antibiotic prophylaxis without differences between the TRAF6 haplotypes (Table 1).

### Characteristics of SBP episodes and outcome according to the *TRAF6* haplotype

We went on to investigate the characteristics of SBP episodes with respect to the *TRAF6* haplotype. There were no significant differences in age at presentation, microbiological culture results, severity of inflammation, organ failure and mortality between patients with a first episode of SBP with the *TRAF6* risk haplotype 2 compared to patients without (Table 3). Short-term survival after a first episode of SBP (Table 3) as well as long-term survival in the overall population were not negatively affected by the presence of the *TRAF6* risk haplotype (Fig. 2D). Independent predictors of overall survival were higher age at inclusion, advanced liver disease as indicated by the MELD score, HCC at inclusion and an episode of SBP at any point according to Cox regression analysis (Table 4).

**Table 3:** Characteristics of the first episode of spontaneous bacterial peritonitis.

	*TRAF6* risk haplotype 2 (N = 40)	TRAF6 non-risk haplotypes (N = 82)	P value
Male sex	26 (65%)	61 (74%)	0.29
Age at SBP (years)	61 (48–68)	57 (51–65)	0.46
AF PMN (×10^9^ cells/l)	1.3 (0.5–4.3)	0.9 (0.4–2.3)	0.14
Bilirubin (µmol/l)	53 (28–100)	52 (23–144)	0.78
INR	1.4 (1.2–1.8)	1.5 (1.3–2.0)	0.24
Creatinine (µmol/l)	128 (85–190)	109 (79–189)	0.59
CRP (mg/l)	50 (27–108)	84 (36–128)	0.11
WBC (×10^9^ cells/l)	8.9 (4.5–13.6)	9.9 (6.9–16.6)	0.10
Hospital-acquired SBP	21 (58%)	42 (52%)	0.55
**Microbial culture result**			
Culture-negative	23 (58%)	51 (62%)	0.38
Gram-negative	7 (18%)	13 (16%)	
Gram-positive	6 (15%)	16 (20%)	
Mixed	1 (3%)	1 (1%)	
N/A	3 (8%)	1 (1%)	
**Renal failure at diagnosis** ^**#**^			
No	25 (63%)	51 (62%)	0.84
Yes	10 (25%)	23 (28%)	
N/A	13 (11%)	8 (10%)	
**Outcome 28 days after diagnosis**			
Survived	33 (83%)	48 (59%)	0.06
Dead	7 (18%)	28 (34%)	
Transplanted	0 (0%)	3 (4%)	
Lost to follow up	0 (0%)	3 (4%)	
Kaplan-Meier estimate ± Std. error	83 ± 6%	65 ± 5%	0.06

Median/IQR or Frequency/Percentage are shown; P values from Mann-Whitney U test, Fisher’s exact test or log-rank test as appropriate.

^#^Kidney failure was defined by serum creatinine >2 mg/dl or the need for renal replacement therapy according to acute-on-chronic liver failure criteria.

Abbreviations: spontaneous bacterial peritonitis (SBP), ascitic fluid (AF), polymorphnuclear cells (PMN), international normalized ratio (INR), C-reactive protein (CRP), white-blood-cell (WBC), N/A: not available.

**Table 4:** Risk factors for death.

	Hazard ratio (95% CI)	*P*-value
(***a***) ***Unadjusted Cox proportional hazard model***		
Age* (per 1-year increase)	1.03 (1.02–1.05)	<**0**.**001**
Male sex	1.07 (0.79–1.44)	0.68
Child Pugh C*	1.70 (1.24–2.32)	<**0**.**001**
MELD* (per 1-point increase)	1.06 (1.04–1.08)	<**0**.**001**
Hepatocellular carcinoma*	2.42 (1.72–3.41)	<**0**.**001**
SBP at any time	1.54 (1.15–2.06)	**0**.**004**
*NOD2* risk variant^#^	0.94 (0.66–1.35)	0.75
*TRAF6* risk haplotype 2	0.82 (0.59–1.15)	0.26
Use of beta blockers*	0.93 (0.70–1.23)	0.93
(***b***) ***Multivariate Cox proportional hazard model***		
Age* (per 1-year increase)	1.04 (1.02–1.05)	<**0**.**001**
Child Pugh C*	Removed from model	n.s.
MELD* (per 1-point increase)	1.07 (1.05–1.09)	<**0**.**001**
Hepatocellular carcinoma*	2.43 (1.71–3.46)	<**0**.**001**
SBP at any time	1.67 (1.25–2.24)	<**0**.**001**

*At inclusion. ^#^Risk variants R702W, G908R and L1007fs.

Abbreviations: confidence interval (CI), spontaneous bacterial peritonitis (SBP), tumor necrosis factor receptor-associated factor 6 (TRAF6), model for end-stage liver disease (MELD), nucleotide-binding oligomerization domain containing 2 (NOD2), not significant (n.s.).

### Functional consequences of *TRAF6* haplotypes on peritoneal immunity

**Figure 3:**
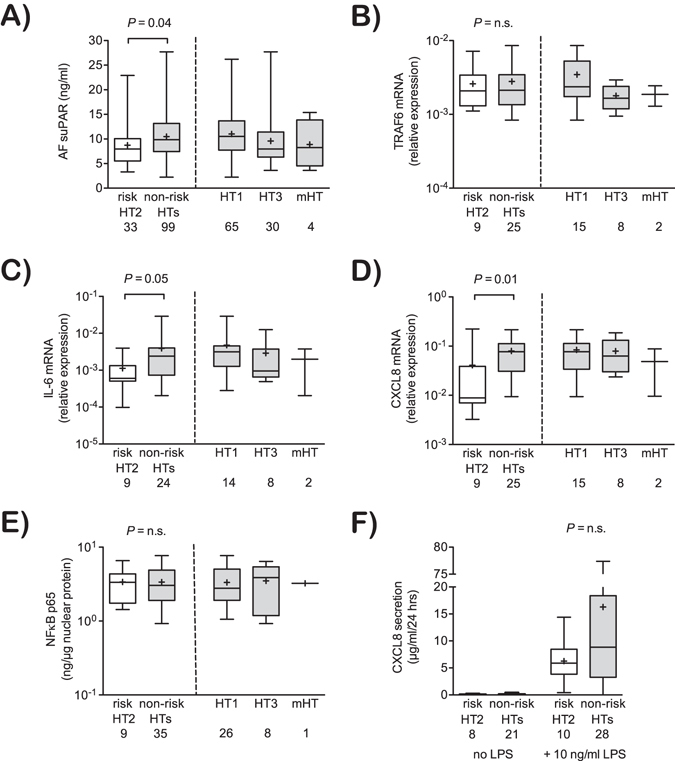
Association of TRAF6 haplotypes with peritoneal immune activation. (**A**) Concentrations of soluble urokinase-type plasminogen activator receptor (suPAR) in non-infected ascites are shown for the different *TRAF6* haplotypes. (**B**–**D**) mRNA expression of *TRAF6*, IL-6 and CXCL8 in freshly isolated peritoneal macrophages are shown for the respective *TRAF6* haplotypes and were expressed relative to the housekeeping gene ß-actin. (**E**) Nuclear concentrations of active NF-κB p65 in freshly isolated peritoneal macrophages are shown for the respective haplotypes. (**F**) Quantification of CXCL8 in supernatants from cultivated primary peritoneal macrophages, which were left unstimulated or were stimulated with LPS (10 ng/ml) for 24 hours. All data are expressed as boxplots (min to max). Non-risk haplotypes (HTs) including HT1, HT3 and the mixed haplotype are indicated (right panels) and were pooled and compared to the risk haplotype HT2 (left panels). Numbers of analyzed samples are indicated. *P* values indicate differences between HT2 and non-risk HTs in Mann-Whitney U test. n.s.: not significant.

To assess whether the *TRAF6* haplotype 2 is associated with altered peritoneal macrophage (PMϕ) function, we first determined the macrophage activation marker soluble urokinase-type plasminogen activator receptor (suPAR) in non-infected ascites^21^. Patients with the *TRAF6* risk haplotype 2 had lower AF suPAR concentrations as compared to other haplotypes (Fig. 3A) indicating a reduced peritoneal immune activation under non-inflammatory conditions in these patients. Although *TRAF6* mRNA expression in isolated PMϕ did not differ between the haplotypes (Fig. 3B), PMϕ from patients carrying the *TRAF6* risk haplotype 2 had lower basal expressions of IL-6 (*P* = 0.05) and CXCL8 (*P* = 0.01) mRNA compared to PMϕ from patients with other haplotypes, confirming a less inflammatory phenotype (Fig. 3C,D). There were no significant differences in nuclear factor (NF)-κB p65 nuclear concentrations (Fig. 3E) and in the concentrations of TRAF6 protein, phosphorylated p38, JNK, and ERK in Western blots in unstimulated patients’ PMϕ (data not shown). After stimulation with 10 ng/ml lipopolysaccharide there was a non-significant trend towards a lower secretion of the neutrophil-recruiting chemokine CXCL8 in patients carrying the *TRAF6* haplotype 2 as compared to other haplotypes (Fig. 3F) suggesting that the LPS-induced release capacity of CXCL8 was not severely compromised in patients with the risk haplotype. Notably, this less inflammatory macrophage phenotype associated with the risk haplotype 2 was only observed in PMϕ but not in circulating monocytes (data not shown).

## Discussion

In this large association study on Caucasian patients with decompensated cirrhosis we demonstrate for the first time an association of genetic germ line haplotype of the *TRAF6* gene with the inflammatory status in peritoneal macrophages and with the risk of developing SBP. A common genetic variant of *TRAF6*, which we named *TRAF6* risk haplotype 2 was found in one out of four patients and conferred an increased risk for SBP as compared either to the wild-type haplotype 1 (OR 1.71; 95% CI 1.04–2.82; *P* = 0.016) or to all patients with a non-risk haplotype (OR 1.95; 95% CI 1.22–3.12; *P* = 0.005). This association remained significant in prospective sensitivity analysis and in multivariate logistic regression after adjustment for confounding factors.

In the absence of SBP, the identified *TRAF6* risk haplotype 2 was accompanied by a less proinflammatory state of peritoneal macrophages as shown by reduced concentrations of suPAR, a soluble marker of peritoneal and systemic immune activation^21^ and lower expression of inflammatory cytokines and chemokines. The lower expression of the neutrophil-recruiting chemokine CXCL8 by PMϕ in patients carrying the TRAF6 risk haplotype 2 suggests an impaired neutrophil recruitment to the peritoneal cavity during viable bacterial translocation resulting in a higher risk for SBP accompanied by a reduced state of peritoneal inflammation. The close association of the degree of systemic inflammation with organ dysfunction and subsequent mortality during bacterial infections in cirrhosis^21–23^, may explain why an increased prevalence of SBP in patients with the *TRAF6* risk haplotype did not translate into increased mortality in our study in presence of adequate treatment. In contrast, there was a non-significant trend towards a less pronounced systemic inflammatory response during SBP and a numerically lower short-term mortality. In the present study, only advanced liver disease as indicated by a higher MELD or lower albumin levels but not total AF protein indicated an increased risk of SBP as described and discussed before^24^.

In our study cohort the minor allele frequencies for the *TRAF6* haplotype-tagging SNPs rs331457 and rs5030419 were 0.16 and 0.15, respectively, well in line with observed minor allele frequencies in healthy European populations of 0.13 (0.10–0.16) and 0.15 (0.11–0.21)^20^. Therefore, the risk to develop cirrhosis *per se* was not significantly influenced by the evaluated *TRAF6* haplotype. Interestingly, the two haplotype-tagging SNPs investigated conferred an opposite effect on the prevalence of SBP, resulting in a genotype at low-risk for SBP (haplotype 3 and mixed haplotype) and at high-risk for SBP (haplotype 2), when compared to the wild type haplotype 1. Notably, we observed immune-modulating effects of the *TRAF6* haplotype in PMϕ but not in circulating monocytes, which could be attributed to (i) different life spans of these cells (long versus short living), (ii) a different compartmentalization (peritoneal cavity versus blood), (iii) the different degree of maturation (differentiated late-stage macrophages versus freshly isolated monocytes), or a combination of these. As we did not observe differences in *TRAF6* expression or mechanistic evidence for deregulated NF-κB activation between patients with different *TRAF6* haplotypes, we cannot attribute whether the observed effects on peritoneal inflammation and infection are direct consequences of altered macrophage signaling or are indirectly mediated by immune cells in other compartments, e.g. in gut-associated lymphatic tissue. In addition, the haplotype tagging SNP rs331457 is in strong linkage disequilibrium with several SNPs in the 3′ untranslated region of the neighboring proline rich 5 like gene (*PRR5L*) and in recombination activating gene 1 (*RAG1*)^19^ (Fig. 1A). Thus, it is also conceivable that the *TRAF6* haplotypes reported herein indicate alterations in *PRR5L* and/or *RAG1* expression affecting immune cell maturation, mechanistic target of rapamycin complex 2 (mTORC2) signaling, and cytokine production^25, 26^. Indeed, the *TRAF6*-associated immune cluster has been implicated in the pathogenesis of inflammatory diseases, such as systemic lupus erythematodes^27, 28^ and rheumatoid arthritis^29^ and in sepsis-associated organ failure^30^.

Our study has two major implications. First, in the light of increasing antimicrobial resistance, individualized approaches that direct antimicrobial prophylaxis only to patients with the highest risk are warranted. *NOD2* genotype-based prophylaxis is currently investigated as a rationale for primary prophylaxis for SBP^31^ but could not be replicated consistently as it may be stronger associated with culture-positive variants of peritonitis^8, 11, 32^. The risk of SBP was the highest in *NOD2* wild type patients with the *TRAF6 risk* haplotype 2, extending the population at risk for genotype-based primary interventions. This may also hint towards a critical role for TRAF6 activation in mediating the susceptibility for SBP, as NOD2-induced ubiquitination of TRAF6 and subsequent inflammatory signaling is altered in the presence of *NOD2* variants^14^. Second, our study provides evidence for a role of *TRAF6* in mediating SBP as a severe complication of decompensated cirrhosis. Therefore, compounds targeting TRAF6 interactions with CD40, which have proven beneficial in animal models of experimental peritonitis and sepsis^33^, may be an attractive target for the prevention of inflammatory and infectious complications in advanced cirrhosis.

## Patients and Methods

### Study design

Patients with decompensated liver cirrhosis who underwent paracentesis at the Jena University Hospital were recruited to study the impact of the *TRAF6* haplotype on the prevalence of SBP and survival. Patients were recruited during three different periods: between 12/2007 and 10/2010 for a genetic association study of SBP and *NOD2* variants as previously reported^8^ (N = 105), between 10/2010 and 01/2013 for a genetic association study of bacterial infections with polymorphisms of the innate immunity (N = 240), and between 06/2013 and 02/2015 to specifically address the impact of *TRAF6* haplotypes on peritoneal and systemic immunity and the risk of SBP (N = 87). The following inclusion criteria had to be fulfilled: 1. Liver cirrhosis defined by clinical, laboratory, or histological criteria; 2. The presence of ascites accessible for diagnostic paracentesis; 3. 18 or more years of age; and 4. The ability to provide informed consent. Exclusion criteria were peritoneal carcinomatosis, secondary peritonitis, and acute pancreatitis. Patients granted written informed consent prior to inclusion. The study conformed to the ethical guidelines of the 1975 Declaration of Helsinki and was approved by the internal review board (Ethics committee of the Jena University Hospital, no. 2880-08/10 and 3683-02/3).

### Samples and patients data

At enrolment, AF and blood samples were obtained. Total and polymorphonuclear (PMN) cell count, protein and albumin concentrations in ascites as well total serum bilirubin, international normalized ratio, creatinine, C-reactive protein, white-blood cell count, platelets, albumin and sodium were determined by routine laboratory analysis. The following variables were collected at study entry: age, gender, etiology of cirrhosis, diagnosis of HCC, Child Pugh class and MELD score. Patients’ medical records were carefully reviewed for a prior documented episode of SBP at study entry. Concomitant medication with antibiotics for primary prophylaxis of SBP (Norfloxacin, Ciprofloxacin, Cotrimoxazole or Rifaximin) and with beta-blockers was either prospectively assessed at study entry for patients included after 10/2013 or collected from medical records for patients included prior to that date. The application of primary antibiotic prophylaxis after inclusion was collected retrospectively from medical records after the end of the study. Patients were followed until death, liver transplantation, loss to follow-up or the end of the study (03/2015). AF PMN counts exceeding 250 cells/µl in the absence of secondary causes were diagnosed as SBP according to international guidelines^34^.

### *TRAF6* and *NOD2* gene analysis

The *TRAF6* haplotype was defined using HapMap data with criteria described in the results section of the manuscript. Genomic DNA was extracted from EDTA whole blood using the DNeasy Blood and Tissue Kit (Qiagen, Hilden, Germany) and adjusted to 20 ng/µl. The *TRAF6* haplotype-tagging SNPs rs331457 and rs5030419 were determined by Fluorescence Resonance Energy Transfer (FRET) melting curve analysis after PCR reaction. Platinum *Taq* DNA Polymerase (Invitrogen, Carlsbad, CA, USA) was used in a 15 µl PCR reaction with 24 ng DNA, 0.2 µM of primers and 80 nM of fluorophore-labeled FRET probes following the manufacturer’s protocol. Genotyping of TRAF6 SNP rs5030445 was performed using a commercial TaqMan SNP Genotyping Assays as described by the manufacturer (ThermoFisher Scientific, Darmstadt, Germany).The NOD2 gene variants R702W (rs2066844), G908R (rs2066845) and 1007fs (rs2066847) were analyzed by TaqMan PCR as described previously^8^. For all primers and probes see Supplementary Table S3.

### Isolation of primary monocytes and peritoneal macrophages

Mononuclear cells from 18 ml peripheral blood and cell pellets collected from 500 ml AF by centrifugation at 800 × *g* for 15 minutes were enriched using density gradient separation with Lympholyte-H (Cedarlane, Burlington, Ontario, Canada). The interphase containing the mononuclear cells was transferred to a new vial, washed and resuspended in PBS supplemented with 2% heat-inactivated fetal bovine serum and 2 mM EDTA. Monocytes and PMϕ were purified by magnetic assisted cell sorting (MACS) with MicroBeads conjugated to anti-human CD14 antibodies (Milteny Biotec, Bergisch Gladbach, Germany). A purity of >95% CD14-positive cells was confirmed using flow cytometry (BD Biosciences, San Jose, CA, USA). Monocytes and PMϕ were snap-frozen for mRNA and protein analysis or cultivated in RPMI 1640 supplemented with 10% (V/V) fetal bovine serum and 1% (W/V) L-Glutamine–Penicillin–Streptomycin solution. *In vitro* stimulation experiments were performed with 10 ng/ml Lipopolysaccharides (LPS) from *E*. *coli K12* (InvivoGen, San Diego, CA, USA). Twenty-four hours after stimulation, supernatant and cells were collected. Cells were lysed using RNA Lysis Buffer (Zymo Research, Irvine, CA, USA) for mRNA.

### Gene expression, cytokine release, and protein expression

RNA was extracted with Zymo Research Quick-RNA MicroPrep Kit with on column DNA digestion (Zymo Research). Purity and concentration was measured by UV absorbance at 260 nm and 280 nm and 500 ng RNA were reverse transcribed with peqGOLD cDNA Synthesis Kit H Plus (PeqLab, Erlangen, Germany) using random hexamer primers. For real-time reverse transcription-PCR (qRT-PCR) Rotor-Gene SYBR Green PCR Kit (Qiagen, Hilden, Germany) was used in a 15 µl reaction with 0.2 µM of forward and reverse primers for the genes encoding for CXCL8, IL-6, TRAF6 and β-actin at least in duplicates (Primer sequences in Supplementary Table S3). For relative quantification data were normalized to the abundance of appropriate ß-actin values by the change-in-threshold (C_T_) method (relative expression = 2^−(C^T^(gene X) − C^T^(ß-actin))^)^35^. For the detection of suPAR in AF and IL-6 and CXCL8 in cell culture supernatants, enzyme-linked immunosorbent assays were performed according to the manufacturer’s instructions in duplicates (suPAR: ViroGates, Birkerød, Denmark; IL-6/CXCL8: RayBiotech Inc., Norcross, GA, USA). TRAF6, p38, JNK, ERK, and active NF-κB protein expression was quantified as described in Supplementary Material and Methods.

### Statistical analysis

Patient’s data at baseline and at first episode of SBP were reported as median/interquartile for continuous data or number/fraction for discrete variables. Differences between groups were tested with Mann-Whitney U-test or Kruskal-Wallis test for continuous and with Fisher´s exact test for discrete variables. Results in two-sided tests with *P* ≤ 0.05 were considered as statistically significant. Risk factors for SBP were determined with binary logistic regression model. Kaplan-Meier methods and log-rank test was used for survival and for SBP incidence analysis. Cox proportional hazard models were used to ascertain the contribution of several factors on survival. For long-term survival analysis, data were right-censored at liver transplantation, loss-to-follow-up, or at the end of the study. For short-term survival after SBP, data were right-censored at liver transplantation, loss-to-follow-up, or after 28 days. For the analysis of the cumulative incidence of a first episode of SBP, data were right-censored at liver transplantation, death, loss-to-follow-up, or end of the study. Candidates for multivariate analysis were variables with *P* ≤ 0.05 in univariate analysis. Statistical calculations were performed with SPSS 21 (IBM, New York, USA) and with GraphPad Prism5 (GraphPad, La Jolla, CA, USA).

### Sample size calculation

Data from the HapMap Project indicated a prevalence of the haplotype tagging SNPs of 25% (homozygous or heterozygous) each. Given an estimated life-time risk of SBP of approximately 33%, a case:control ratio of 1:3 per SNP, a type I error probability of 0.025 (two SNPs), and an estimated true odds ratio of 2.5 for an association of one of the SNPs with SBP^9^, this study was planned to include a minimum of 268 patients to be able to reject the null hypothesis that this odds ratio equals 1 with power of 80% or more.

## Electronic supplementary material

Supplement
