# Supplementary material for: Genetic variants of TRAF6 modulate peritoneal immunity and the risk of spontaneous bacterial peritonitis in cirrhosis: A combined prospective-retrospective study
Source: Sci Rep. 2017 Jul 7;7:4914. doi: 10.1038/s41598-017-04895-z (PMC5501819; doi:10.1038/s41598-017-04895-z)
Supplement: Supplementary file 1 — Supplement [file 41598_2017_4895_MOESM1_ESM.doc]

**Supplementary material to the manuscript**

**Genetic variants of TRAF6 modulate peritoneal immunity and the risk of spontaneous bacterial peritonitis in cirrhosis: A combined prospective-retrospective study**

Martina Mai (1,2) martina.mai@med.uni-jena.de

Sven Stengel (2) sven.stengel@med.uni-jena.de

Eihab Al-Herwi (2) eihab.al-herwi@med.uni-jena.de

Jack Peter (2) jack.peter@med.uni-jena.de

Caroline Schmidt (3) caroline.schmidt@med.uni-jena.de

Ignacio Rubio (1,3) ignacio.rubio@med.uni-jena.de

Andreas Stallmach (1,2) andreas.stallmach@med.uni-jena.de

Tony Bruns (1,2) tony.bruns@med.uni-jena.de

1. The Integrated Research and Treatment Center for Sepsis Control and Care (CSCC), Jena University Hospital, Friedrich Schiller University of Jena, Jena, Germany

2. Department of Internal Medicine IV (Gastroenterology, Hepatology, and Infectious Diseases), Jena University Hospital, Friedrich Schiller University of Jena, Jena, Germany

3. Institute of Molecular Cell Biology, Center for Molecular Biomedicine (CMB), Jena University Hospital, Friedrich Schiller University of Jena, Jena, Germany

**Content:**

Supplementary Material and Methods

Supplementary Tables S1-S2

Supplementary Material and Methods

Western Blots

Twenty-five microgram of cellular protein were subjected to sodium dodecyl sulfate polyacrylamide gel electrophoresis (SDS-PAGE), transferred to polyvinylidene fluoride (PVDF) membranes (Merck Millipore, Darmstadt, Germany) for immunoblotting. Western blot analysis was performed with primary antibodies and the corresponding horseradish peroxidase-conjugated anti-rabbit or anti-mouse IgG secondary antibodies (KPL, Inc., Gaithersburg, MD, USA). Chemiluminescence was performed with Western Lightning ECL (PerkinElmer Inc., USA) and was detected in the FujiFilm LAS-3000 imaging system (FujiFilm, USA). The antibodies used were as follows: anti-p38 MAPK antibody (Cell Signaling, #9212), anti-Phospho-p38 MAPK (Thr180/Tyr182) (3D7) antibody (Cell Signaling, #9215), anti-Phospho-p44/42 MAPK (ERK1/2) (Thr202/Tyr204) (E10) antibody (Cell Signaling, #9106), anti p44/42 (ERK1/2) (137F5) antibody (Cell Signaling, #4695), anti-JNK/SAPK1 (37/pan-JNK/SAPK1) antibody (BD Biosciences, San Jose, CA, USA, #610627), anti-JNK/SAPK (pT183/pY185) antibody (BD Biosciences, #612540) and anti-β-Actin (AC-15) antibody (Sigma-Aldrich, St. Louis, MO, USA, #A5441).

ELISA –based analysis of active Nuclear Factor kappa B p65

For analysis of active NF-κB p65 nuclear proteins were extracted with Nuclear Extract Kit (Active Motif, La Hupe, Belgium) from isolated PMϕ. Protein concentration was determined with Bradford Microassay (Carl Roth, Karlsruhe, Germany) and absorption was detected with microplate reader Infinite Pro F500 (Tecan, Männerdorf, Switzerland). Active p65 was analysed with TransAM NFκB p65 DNA-binding ELISA Kit (Active Motif, La Hupe, Belgium). Recombinant p65 protein (Active Motif, La Hupe, Belgium) was used to prepare a standard curve. All procedures were performed according manufacturers protocols.

**Supplementary Table S1. Baseline characteristics in patients without hepatocellular carcinoma**

| **rs331457 rs5030419** | **All patients   (N=367)** | **Haplotype 1**  **WT (G) WT (C)  (N=175)** | **Haplotype 2**  **MUT (G>A) WT (C)  (N=92)** | **Haplotype 3**  **WT (G) MUT (C>G) (N=85)** | **Mixed Haplotype**  **MUT (G>A) MUT (C>G) (N=15)** | **P value^#^** |
| --- | --- | --- | --- | --- | --- | --- |
| **Male sex** | 252 (69%) | 118 (67%) | 60 (65%) | 62 (73%) | 12 (80%) | 0.56 |
| **Age at inclusion** | 58 (51-68) | 59 (53-68) | 58 (47-67) | 57 (50-69) | 55 (52-65) | 0.39 |
| **Alcoholic cirrhosis** | 281 (77%) | 131 (75%) | 71 (77%) | 66 (78%) | 13 (87%) | 0.82 |
| **NOD2 risk allele** | 73 (20%) | 35 (20%) | 21 (23%) | 16 (19%) | 1 (7%) | 0.59 |
| **Child Pugh C** | 241 (66%) | 119 (68%) | 60 (65%) | 55 (65%) | 7 (47%) | 0.42 |
| **MELD** | 17 (12-22) | 17 (12-22) | 19 (12-22) | 17 (12-23) | 13 (11-17) | 0.14 |
| **AF protein (g/l)** | 12.9 (8.0-19.8) | 13.6 (8.8-19.5) | 10.0 (8.0-17.6) | 11.0 (8.0-20.8) | 22.0 (13.4-37.3) | **0.011** |
| **Bilirubin (µmol/l)** | 42 (21-97) | 41 (21-100) | 46 (25-91) | 46 (17-115) | 35 (23-56) | 0.78 |
| **INR** | 1.5 (1.2-1.7) | 1.5 (1.2-1.8) | 1.4 (1.2-1.7) | 1.5 (1.2-1.8) | 1.3 (1.1-1.6) | 0.39 |
| **Creatinine (µmol/l)** | 97 (66-149) | 90 (67-149) | 104 (61-160) | 105 (68-144) | 82 (67-135) | 0.76 |
| **CRP (mg/l)** | 32 (16-56) | 31 (15-56) | 37 (18-61) | 32 (17-52) | 28 (15-61) | 0.85 |
| **WBC (10^9^ cells/l)** | 7.4 (5.0-11.1) | 7.1 (5.3-10.3) | 7.0 (4.6-13.0) | 8.9 (5.5-12.3) | 5.7 (3.9-10.2) | 0.31 |
| **Platelets (10^9^ cells/l)** | 129 (83-187) | 138 (87-199) | 132 (81-189) | 114 (79-183) | 96 (66-173) | 0.43 |
| **Albumin (g/l)** | 24 (20-29) | 24 (20-28) | 23 (20-28) | 25 (20-29) | 28 (19-35) | 0.74 |
| **Sodium (mmol/l)** | 136 (132-139) | 136 (131-139) | 136 (132-138) | 135 (132-139) | 136 (132-141) | 0.59 |
| **SBP at any point SBP recurrence** | 106 (29%) 18 (17%) | 49 (28%) 6 (12%) | 37 (40%) 7 (19%) | 19 (22%) 5 (26%) | 1 (7%) 0 | **0.011** 0.45 |
| **Primary prophylaxis^¶^** | 61 (17%) | 28 (16%) | 15 (17%) | 16 (19%) | 2 (13%) | 0.94 |

Median/IQR or Frequency/Percentage are shown; ^#^P values from from Kruskal Wallis test or Fisher’s exact test as appropriate. ^¶^ Comprising quinolones, cotrimoxazole and rifaximin. Abbreviations: nucleotide-binding oligomerization domain-containing protein 2 (NOD2), model for end-stage liver disease (MELD), ascitic fluid (AF), polymorphnuclear cells (PMN), international normalized ratio (INR), C-reactive protein (CRP), white-blood-cell (WBC), spontaneous bacterial peritonitis (SBP)

**Supplementary Table S2. Primers and probes used for genotyping and mRNA expression analysis**

| Primer | Sequence |
| --- | --- |
| **CXCL8 forward** | TCCTGATTTCTGCAGCTCTGT |
| **CXCL8 reverse** | AAATTTGGGGTGGAAAGGTT |
| **IL6 forward** | AGTGAGGAACAAGCCAGAGC |
| **IL6 reverse** | GTCAGGGGTGGTTATTGCAT |
| **TRAF6 forward** | TGGCATTACGAGAAGCAGTG |
| **TRAF6 reverse** | GTTCCATCTTGTGCAAACAACC |
| **ß-actin forward** | CATGTACGTTGCTATCCAGGC |
| **ß-actin reverse** | CTCCTTAATGTCACGCACGAT |
| **TRAF6 rs331457 forward** | TCTCCACACTGAGCATTTGG |
| **TRAF6 rs331457 reverse** | ACAGTAAGTGTTCACTAAGCATC |
| **TRAF6 rs331457 anchor** | LC610 -TTCCCTTCAGCTCAGGATACTTCAAG- PH |
| **TRAF6 rs331457 sensor** | CACTGAACTGCTAGCCATACT- FL |
| **TRAF6 rs5030419 forward** | CAGCTGAGTTAGGACTGTC |
| **TRAF6 rs5030419 reverse** | ATGATAGCGGCCAAGTTCAC |
| **TRAF6 rs5030419 anchor** | LC640 -CAGGTTCCATGTAGAAACGAGAAAAACT- PH |
| **TRAF6 rs5030419 sensor** | CTATTACTGAATGTCTTGTTAC- FL |
| **NOD2 R702W forward** | TTCCTGGCAGGGCTGTTGTC |
| **NOD2 R702W reverse** | AGTGGAAGTGCTTGCGG |
| **NOD2 R702W probe 1** | FAM-CCTGCTCCGGCGCCAGGC-TAMRA |
| **NOD2 R702W probe 2** | VIC-CCTGCTCTGGCGCCAGGCC-TAMRA |
| **NOD2 G908R forward** | ACTCACTGACACTGTCTGTTGACTCT |
| **NOD2 G908R reverse** | AGCCACCTCAAGCTCTGGTG |
| **NOD2 G908R probe 1** | FAM-TTTTCAGATTCTGGGGCAACAGAGTGGGT-TAMRA |
| **NOD2 G908R probe 2** | VIC-TTCAGATTCTGGCGCAACAGAGTGGGT-TAMRA |
| **NOD2 1007fs forward** | GTCCAATAACTGCATCACCTACCTAG |
| **NOD2 1007fs reverse** | CTTACCAGACTTCCAGGATGGTGT |
| **NOD2 1007fs probe 1** | FAM-CCCTCCTGCAGGCCCTTGAAAT-TAMRA |
| **NOD2 1007fs probe 2** | VIC-CCTCCTGCAGGCCCCTTGAAA-TAMRA |
